# The efficacy of small molecule anti-angiogenic drugs in previously treated Thymic carcinoma

**DOI:** 10.1186/s12885-022-10448-z

**Published:** 2023-01-05

**Authors:** Yelan Guan, Xiaodong Gu, Jinfei Si, Jing Xiang, Jingwen Wei, Yue Hao, Wenxian Wang, Yan Sun

**Affiliations:** 1 Wenzhou Medical University, Wenzhou, 325035 Zhejiang China; 2 Department of Medical Oncology, The Cancer Hospital of the University of Chinese Academy of Sciences (Zhejiang Cancer hospital), Hangzhou, 310022 Zhejiang China; 3 Institute of Basic Medicine and Cancer (IBMC), Chinese Academy of Sciences, Hangzhou, 310022 Zhejiang China; 4 The Second Clinical Medical College of Zhejiang Chinese Medical University, Hangzhou, 310053 Zhejiang China

**Keywords:** Thymic carcinoma, Apatinib, Anlotinib, Anti-angiogenic drugs, Efficacy

## Abstract

**Background:**

Antiangiogenic drugs have shown initial efficacy in the treatment of advanced thymic carcinomas (TCs); however, data are limited. In this study, we provide real-world data relating to the efficacy of antiangiogenic drugs for the treatment of patients with TCs.

**Methods:**

We retrospectively collected data on clinical progress after first-line chemotherapy in TCs patients who were treated with small molecule antiangiogenic drugs at our institution between January 2010 and December 2021. Tumor response was evaluated according to version 1.1 of the Response Evaluation Criteria in Solid Tumors. Progression free survival and overall survival were calculated using the Kaplan-Meier method.

**Results:**

Of the 17 patients enrolled, 13 (76.5%) received apatinib and four (23.5%) anlotinib monotherapy with an objective response rate of 23.5%. Eleven (64.7%) patients had stable disease. The median follow-up period was 46.0 months (95% confidence interval [CI], 33.0–59.0 months). The median progression survival and overall survival were 7.9 months (95% CI, 6.5–9.3) and 47.0 months (95% CI, 35.4–58.6), respectively. In the 13 patients receiving apatinib, the median PFS was 7.0 months (95% CI, 5.0–9.0), compared with 8.0 months (95% CI, 2.7–13.3 months) for patients in the anlotinib group (*P* = 0.945). The most common grade 3 adverse events (AEs) were hypertension (*n* = 3, 23.1%), followed by proteinuria and hand-foot syndrome (HFS, *n* = 2, 15.4%). There were no grade 4 AEs although eight patients (47.1%) required mid-course discontinuation.

**Conclusion:**

For refractory TCs, small molecule antiangiogenic drugs are efficacious as second- or post-line treatments. The toxicity of antiangiogenic therapy is manageable.

**Supplementary Information:**

The online version contains supplementary material available at 10.1186/s12885-022-10448-z.

## Background

Thymic carcinomas (TCs) are aggressive mediastinal malignant tumors with an annual incidence of 1.3–3.2 per million [1, 2]. Patients with early-stage TCs often choose to undergo complete surgical resection and postoperative adjuvant radiotherapy. Paclitaxel combined with carboplatin is the recommended first-line treatment for patients with according to the National Comprehensive Cancer Network guideline with an overall response rate (ORR) of 22–36%. Unfortunately, advanced TCs show a limited response to chemotherapy and patients with TCs lack an effective second-line regimen after disease progression. Chemotherapy has shown a median progress-free survival time (mPFS) of 4.3–7.6 months as a second-line therapy in patients with recurrent TCs according to several prospective phase 2 studies [3–6]. In recent years, targeted drugs and immune checkpoint inhibitors (ICIs) have made some progress for the treatment of advanced thymic carcinomas. For previously treated TCs patients, the mPFS and ORR of ICIs monotherapy (pembrolizumab and nivolumab) were 3.8–6.1 months and 0–22.5%, respectively [7–9]. Clinical studies on second- and posterior treatment options for TCs are numerous, although most are limited to small samples and evidence from randomized controlled trial is lacking. As such, for patients with TCs who have progressed after first-line platinum-containing chemotherapy, standard second- or post-line treatment regimens have not been confirmed.

Over recent years, the efficacy of angiogenesis therapy in advanced non-small cell lung cancer, soft tissue sarcoma, and radioactive iodine-refractory differentiated thyroid cancer has been recognized. Clinical studies of antiangiogenic drugs in the treatment of metastatic TCs are gaining attention. For example, lenvatinib, sunitinib, bevacizumab and regorafenib have all showed some antitumor activity in patients with previously treated TCs [10–15]. Lenvatinib is known to exhibit the highest ORR of 38% as the second-line treatment in metastatic TCs patients [10].

Vascular endothelial growth factor receptor-2 (VEGFR2) and platelet-derived growth factor receptor-α (PDGFR-α) are known to be activated and involved in the growth and progression of TCs [16–19]. However, evidence on the efficacy of anlotinib and apatinib in the treatment of advanced TC is limited. Anlotinib is a newly developed oral small-molecule tyrosine kinase inhibitor (TKI) that targets VEGFR1, VEGFR2/KDR, VEGFR3, c-Kit, PDGFR-α, and the fibroblast growth factor receptor (FGFR1, FGFR2, and FGFR3). Furthermore, anlotinib can inhibit both tumor angiogenesis and tumor cell proliferation [20, 21]. Apatinib exerts its antigenic effects by inhibiting VEGFR-induced proliferation and migration of endothelial cells via the selectively targeting of VEGFR2 [22].

Based on this background, we explored the efficacy and toxicity of small molecule antiangiogenic drugs in previously treated patients with advanced TCs in the real-word.

## Methods

### Patients

All patient information was obtained from the electronic medical records system of Zhejiang Cancer Hospital (Hangzhou, Zhejiang). Patients with pathologically documented metastatic TCs (stage IVA or IVB defined by the Masaoka-Koga classification) after failure of first-line or multiline chemotherapy who used small molecule antiangiogenic drugs in the next were included in this study from January 2010 to December 2021. The inclusion criteria were as follows: (1) Eastern Cooperative Oncology Group (ECOG) performance status 0 or 1, (2) at least one measurable target lesion defined by the Response Evaluation Criteria in Solid Tumors (RECIST) version 1.1 and (3) received at least two cycles of small molecule antiangiogenic therapy after progression on a first-line platinum-based chemotherapy. The exclusion criteria were: (1) patients received immunotherapy or other targeted agents other than small molecule antiangiogenic drugs, (2) patients had an active autoimmune disease, and (3) patients had malignant tumors of other sites. Recurrence or metastases were performed evaluated by chest computed tomography (CT) scan, abdominal brain CT, or bone scans.

The study protocol was approved by the Institutional Ethics Committee at Zhejiang Cancer Hospital (No. IRB-2022-62). Individual patient consent was not required for this study.

### Responses assessments

Patients received oral apatinib 250–500 mg per day or oral anlotinib 12 mg per day for 2 weeks, with 1 week off; each cycle was 3 weeks in length. Tumor responses were assessed every two cycles or evaluated early when significant signs of progression appeared. Objective tumor responses were according to RECIST 1.1, and include complete response (CR), partial response (PR), stable disease (SD), and progressive disease (PD). Treatment related adverse events (AEs) grade as assessed by the National Cancer Institute Common Terminology Criteria for Adverse Events (CTCAE) version 5.0.

### Statistical analysis

Data analyses were conducted using SPSS version 25 (SPSS Inc., Chicago, IL, USA) software. *T* test was used for normally distributed variables such as age between groups. The non-parametric rank sum test was used for continuous variables between groups that were not normally distributed. Other categorical variables were compared using Fisher’s exact Chi-squared test. *P* < 0.05 was considered significant. Survival analysis was calculated using the Kaplan-Meier method. PFS encompassed the time from the first cycle of antiangiogenic drugs therapy to documented progression or death from any cause. Overall survival (OS) was defined as the time diagnosed with advanced thymic carcinoma to death from any cause or last follow-up. COX regression was used for the univariate analysis of PFS and OS and significant variables (*P* < 0.05) were included in subsequent multivariate analysis. The median follow-up period was 46.0 months (95% confidence interval [CI], 33.0–59.0 months, adverse Kaplan-Meier estimated) and the last follow-up was the 17th of April 2022.

## Results

### Patients

A total of 17 patients were enrolled between January 2010 and December 2021, excluding those for whom PFS data were not available. The baseline characteristics of all patients were summarized in Table 1. The median age was 59 years (range, 35–73 years). Ten (58.8%) of the 17 patients were male. All subjects had an ECOG performance status of 0 or 1. Twelve patients had never smoked, and five patients had smoked before or were still smoking. Squamous cell thymic carcinoma was the most common type of pathology, accounting for 58.8% (10/17) of the subjects. Three cases had undifferentiated carcinoma, whereas the specific pathological type of the remaining four cases was unknown. Only one patient had a Masaoka-koga staging of IVA, whereas others were staged at IVB. The most common site of distant metastases was the lungs (76.5%, 13/17), followed by liver and bone (both 35.3%, 6/17). In addition, one (5.9%) patient had adrenal metastasis. Furthermore, of the 17 patients, ten (58.8%) had received one line of systemic chemotherapy at most, whereas seven (41.2%) had received two or more lines of chemotherapy. Thirteen patients (76.5%) were treated with apatinib and another four patients (23.5%) accepted anlotinib treatment.

**Table 1:** The characteristics of patients

Characteristics, n (%)	Total (***n*** = 17)	Apatinib (***n*** = 13)	Anlotinib (***n*** = 4)	***P***
Age, median(range), yr	59 (35–73)	60 (35–73)	58 (49–66)	0.881*
Sex, male/female	10/7 (58.8 vs. 41.2)	7/6 (53.8 vs. 46.2)	2/2 (50.0 vs. 50.0)	1.000**
ECOG PS				1.000**
0	6 (35.3)	5 (38.5)	1 (25.0)	
1	11 (64.7)	8 (61.5)	3 (75.0)	
Smoking status				0.538**
Never	12 (70.6)	10 (76.9)	2 (50.0)	
Former or current	5 (29.4)	3 (23.1)	2 (50.0)	
WHO histology				0.307**
Squamous cell carcinoma	10 (58.8)	6 (46.2)	4 (100.0)	
Undifferentiated carcinoma	3 (17.6)	3 (23.1)	0 (0.0)	
Unavailable	4 (23.5)	4 (30.8)	0 (0.0)	
Masaoka-Koga stage				1.000**
IVA	1 (5.9)	1 (7.7)	0 (0.0)	
IVB	16 (94.1)	12 (92.3)	4 (100.0)	
Number of prior treat-line				1.000**
1	10 (58.8)	8 (61.5)	2 (50.0)	
>1	7 (41.2)	5 (38.5)	2 (50.0)	
Surgical Procedures				0.541**
Yes	3 (17.6)	3 (23.1)	0 (0.0)	
No	14 (82.4)	10 (76.9)	4 (100.0)	
Lung metastasis				1.000**
Yes	13 (76.5)	10 (76.9)	3 (75.0)	
No	4 (23.5)	3 (23.1)	1 (25.0)	
Liver metastasis				0.584**
Yes	6 (35.3)	4 (30.8)	2 (50.0)	
No	11 (64.7)	9 (69.2)	2 (50.0)	

*p value calculated by t test

**p value calculated by Fisher’s exact test

### Response and survival analysis

**Fig. 1:**
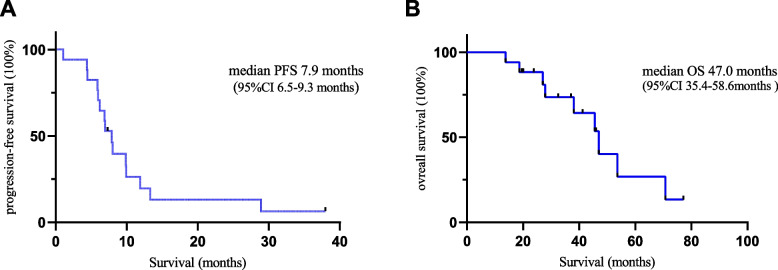
Survival curve of 17 patients using the Kaplan-Meier method. **A** progression-free survival (PFS), **B** overall survival (OS)

As shown in Table 2, four of the 17 patients had a partial response and 11 had stable disease, with an ORR of 23.5% and a disease control rate (DCR) of 88.2%. In the total population, the mPFS was 7.9 months (95% CI, 6.5–9.3 months) and the median OS (mOS) was 47.0 months (95% CI, 35.4–58.6 months) (Fig. 1). Of the patients who received apatinib treatment, four patients (4/13, 30.8%) achieved a partial response, seven patients (53.8%) had stable disease, and two patients (15.4%) showed disease progression. However, none of the patients who received anlotinib had an objective response, probably because of the small sample size. All patients (4/4,1005.0%) had stable disease in the anlotinib group. ORR did not differ significantly between subgroups (*P* = 0.519).

**Table 2:** The efficacy of small molecule antiangiogenic drugs in previously treated thymic carcinomas

	Total (***n*** = 17)	Apatinib (***n*** = 13)	Anlotinib (***n*** = 4)	***P***
Partial response	4 (23.5)	4 (30.8)	0 (0.0)	
Stable response	11 (64.7)	7 (53.8)	4 (100.0)	
Progressive disease	2 (11.8)	2 (15.4)	0 (0.0)	
Overall response rate, %	23.5	30.8	0.0	0.519*
mPFS(95%CI), mo	7.9 (6.5–9.3)	7.0 (5.0–9.0)	8.0 (2.7–13.3)	1.000**
mOS(95%CI), mo	47.0 (35.4–58.6)	47.0 (43.7–50.2)		0.076***

*p value calculated by Fisher’s exact test

**p value calculated by Wilcoxon rank sum test

***p value calculated by t test

**Fig. 2:**
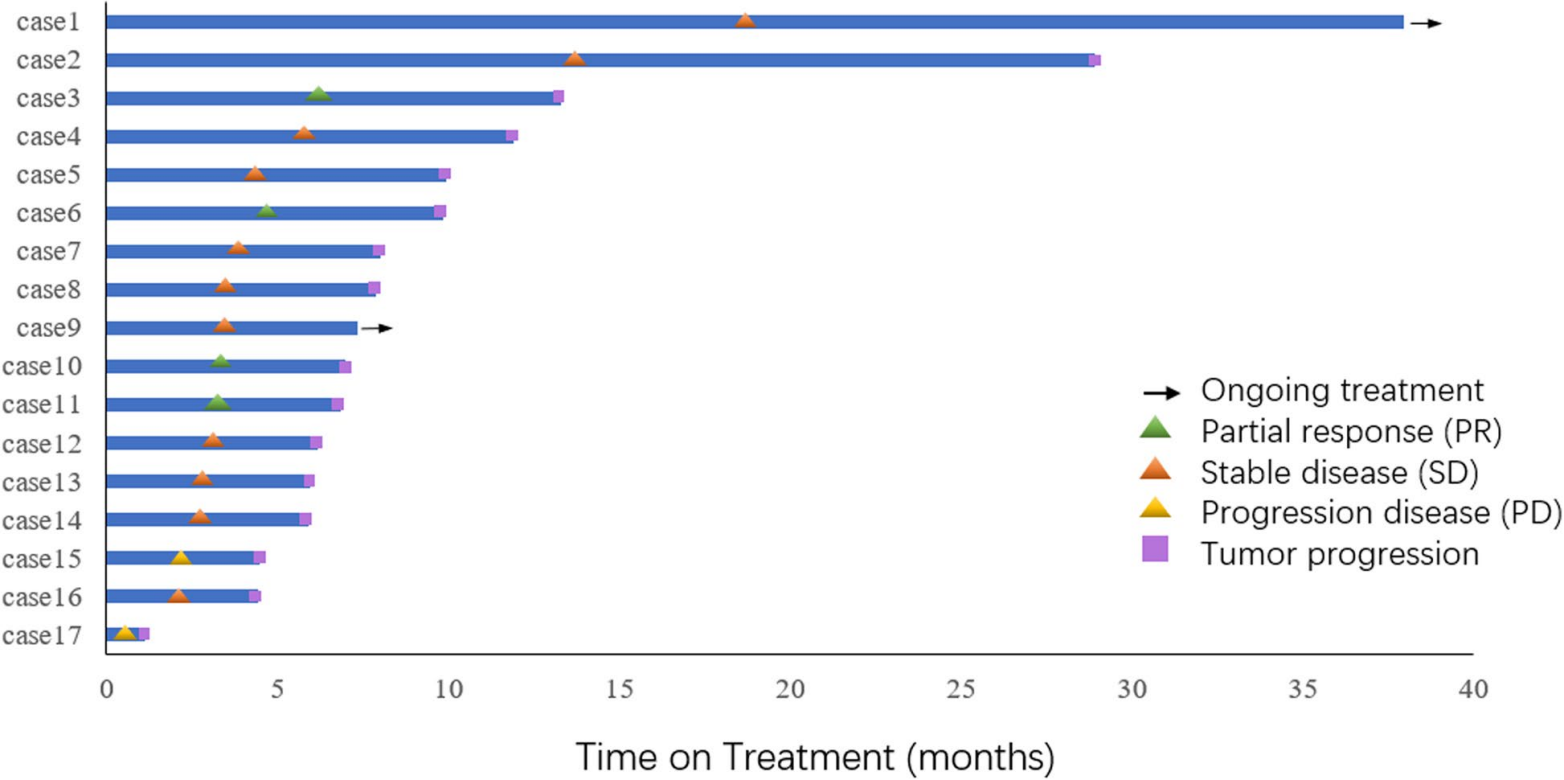
Swimmer plots of small molecule antiangiogenic drugs for previously treated patients with advanced thymic carcinomas

Figure 2 shows the time of discontinuation for apatinib and anlotinib. There were two patients (one apatinib and one anlotinib) still receiving treatment at the data cut-off point. The mPFS in the apatinib and alotinib groups were 7.0 months (95% CI, 5.0–9.0 months) and 8.0 months (95% CI, 2.7–13.3 months), respectively (*P* = 0.945). The mOS for patients receiving apatinib was 47.0 (95% CI, 43.7–50.2) months, whereas mOS for the group receiving anlotinib could not be determined due to the small sample size (*P* = 0.076). There was no significant difference in the therapeutic efficacy when compared between the two drugs. Eight patients were still alive at the data cut-off date (17th of April 2022). In the univariate COX regression analysis of PFS, only performance status score (0 vs. 1) was statistically significant (*P =* 0.026; HR = 4.531; 95% CI, 1.20–7.16) (see Supplementary Table 1).

In addition, all patients had previously received paclitaxel combined with platinum (paclitaxel plus carboplatin, paclitaxel plus cisplatin, or docetaxel plus cisplatin) as the first-line chemotherapy regimen. In total, 11 of the 17 (64.7%) patients received the paclitaxel plus cisplatin regimen, 4/17 (23.5%) patients received the paclitaxel plus cisplatin regimen and one patient (5.9%) received the docetaxel plus cisplatin regimen, respectively. Sixteen patients were evaluated. Of these, five patients had a partial response, ten patients had stable disease, and one patient showed disease progression. The mPFS for first-line platinum chemotherapy was 7.1 months (95% CI, 5.1–9.2 months).

### Toxicity

The AEs observed in all patients at any grade was summarized in Table 3. There were four patients which could not be assessed due to incomplete information. The most common grade 1–2 AEs were hand-foot syndrome (HFS), blood toxicity, and proteinuria (both 5/13, 38.5%). The most common grade 3 AEs was hypertension (3/13, 23.1%), followed by HFS and blood toxicity (both 2/13, 15.4%). No grade 4 or 5 toxicities occurred. However, in the total population, eight patients (47.1%) required suspension of the drug during treatment due to intolerable toxic reactions. Fortunately, eventually all patients who needed to stop treatment resumed treatment. No patients died due to toxic reactions.

**Table 3:** Treatment-related adverse events at any grade in patients

	***N***, grade 1–2, %	***N***, grade 3, %
Hand-foot syndrome	5 (38.5)	2 (15.4)
Fatigue	2 (15.4)	1 (7.7)
Mouth ulcers	4 (30.8)	1 (7.7)
Blood toxicities	5 (38.5)	1 (7.7)
Proteinuria	5 (38.5)	2 (15.4)
Diarrhea	3 (23.1)	0 (0.0)
Hypertension	3 (23.1)	3 (23.1)
Elevated creatinine	1 (7.7)	0 (0.0)

Four patients could not be evaluated due to incomplete medical record information; Blood toxicities include decreased neutrophil count, decreased platelet count, decreased hemoglobin count and decreased platelet count; No grade 4 or 5 AEs occurred

## Discussion

To the best of our knowledge, this is the first real-world retrospective study of small molecule antiangiogenic drugs for previously treated thymic carcinoma in China. This research involved the largest sample size of small molecule antiangiogenic drugs for advanced TCs. Our analysis showed that apatinib and anlotinib exhibited considerable antitumor activity and durable responses in advanced TCs.

We summarized the phase II studies of small molecule antiangiogenic drugs in advanced thymic carcinomas (Table 4). Four phase II studies have explored the efficacy of antiangiogenic drugs (lenvatinib, sunitinib, apatinib, and regorafenib) for the treatment of advanced TCs. The *National Comprehensive Cancer Network (NCCN) Guidelines Version 2.2022 Thymomas and Thymic Carcinomas* recommended sunitinib with an ORR of 26.0% as the second-line treatment regimen for advanced TCs [11]. Another phase II trial of lenvatinib for 42 patients with advanced TCs, reported the best ORR of 38.0% (95% CI, 25.6–52.0, *p* < 0.0001), a mPFS of 9.3 (95% CI, 7.7–13.9) months, and a DCR of 95.0%. Similarly, the most common grade 3 AEs were hypertension (64.0%) and hand-foot syndrome (7.0%). Song et al. [23] previously evaluated the efficacy of apatinib in the treatment of advanced TCs, with an ORR, DCR, mPFS and mOS of 20.0 and 73.0%, 6.1 months (95%CI, 2.6–9.6 months) and 24.0 months (95%CI, 16.1–not evaluable (NE) months), respectively. Another phase II study [15] explored the efficacy of regorafenib monotherapy in eight patients with TCs as the second-line regimen. These authors reported a mPFS of 9.2 months (95% CI, 0.9–34.0 months) and a mOS of 20.1 months (95% CI, 0.9–NE) months, although the ORR was 0.0% according to the RECIST criteria.

**Table 4:** Phase II study of small molecule antiangiogenic agents in advanced thymic carcinomas

Researcher	Year	Study	Drug	Number	mPFS	mOS	ORR
Thomas [11]	2015	Phase II	Sunitinib	25TC	7.2	NA	26.0%
Sato [10]	2020	Phase II	lenvatinib	42TC	9.3	NA	38.0%
Song [23]	2022	Phase II	Apatinib	15TC	6.1	24.0	20.0%
Perrino [15]	2022	Phase II	Regorafenib	8TC	9.2	20.1	0.0%

*mPFS* median progression-free survival, *mOS* median overall survival, *ORR* overall response rate, *NA* not available

In addition, Yudong et al. [24] (2018) reported a case of advanced thymic squamous cell carcinoma with EGFR exon 20 insertion that was effectively treated with apatinib after the failure of multiple lines of chemotherapy. The patient had received a partial response after 5 months of treatment and the PFS was 10.0 months. Similarly, a patient with advanced thymic squamous carcinoma with EGFR 20 exon insertion was successfully treated with apatinib and anlotinib after failure of previous multiline therapy [25]. This study showed that after reaching a PFS of 13.0 months with apatinib treatment, the patient was switched to anlotinib due to adverse effects. The PFS of anlotinib was 23.0 months and the OS was 6 years. After treatment with apatinib failed, anlotinib was able to control the patient’s mediastinal mass and all adverse effects were tolerated. He et al. [26] (2018) also reported a case of partial response following daily treatments with apatinib for advanced TC; the PFS was 6.3 months and drug-related toxicities were tolerable.

In contrast with previous studies, our present research included two small molecule TKI drugs for the treatment of TCs. Certainly, the ORR of this study was consistent with that of the four phase II studies. We observed a mOS of 47.0 (95%CI, 35.4–58.6 months) after a median follow-up of 46 months and there were eight (47.1%) surviving patients at the last follow-up date. In addition, we did not identify any significant difference in terms of efficacy between anlotinib and apatinib. Head-to-head studies of anlotinib and apatinib in advanced thymic carcinomas are now expected.

VEGF is involved in the development of normal blood vessel networks in the thymus [16]. Several previous studies have shown that VEGFR1 and VEGFR2 are highly expressed in thymic carcinomas. Angiopoietin 1 and VEGF are known to be the most up-regulated growth factors in TCs [17, 18, 27].

Our real-world data also reported the efficacy of antiangiogenic drugs for the treatment of advanced TCs. Consequently, small molecule angiogenesis therapy may represent a new treatment option for recurrent or metastatic advanced thymic carcinoma patients. TKIs have been observed to alter the tumor microenvironment by inducing immune microenvironment remodeling [28, 29]. Small molecule antiangiogenic agents combined with ICIs have shown good efficacy for a variety of solid tumors [30, 31]. Thus, novel combinations of small molecule antiangiogenic drugs with ICIs in patient with recurrent TCs need to be explored further. For example, a clinical trial of pembrolizumab in combination with sunitinib for advanced TCs (NCT03463460) is ongoing.

There are some limitations to our study that need to be considered. Firstly, this was a single-center, retrospective study and therefore retrospective nature of this study could have influenced some of the results. Second, only a limited number of cases were included in our study due to the fact that thymic tumors are relatively rare. Finally, this study was also limited by the small sample size in subgroup analysis.

## Conclusion

In the real world, antiangiogenic drugs have shown some efficacy in advanced TCs. The mode of combination small molecule antiangiogenic drugs with chemotherapy or immunologic agents needs to be further explored in the future.

## Supplementary Information

**Additional file 1: Supplementary Table 1.** Univariate COX regression analysis of PFS and OS.

## Data Availability

The datasets used and/or analyzed in the current study are available from the corresponding authors on reasonable request.

## Abbreviations

TCs: Thymic carcinomas
WHO: World Health Organization
NCCN: National Comprehensive Cancer Network
ECOG: Eastern Cooperative Oncology Group
HFS: Hand-foot syndrome
VEGFR2: Vascular endothelial growth factor receptor-2
PDGFR-α: Platelet-derived growth factor receptor-α
TKI: Tyrosine kinase inhibitor
FGFR1: Fibroblast growth factor receptor
ICIs: Immune checkpoint inhibitors
mPFS: median progression-free survival
mOS: median overall survival
CI: Confidence interval
AEs: Adverse events
CR: Complete response
PR: Partial response
SD: Stable disease
PD: Progressive disease
DCR: Disease control rate
ORR: Objective response rate
HR: Hazard ratio
RECIST: Response Evaluation Criteria in Solid Tumors
CTCAE: Common Terminology Criteria for Adverse Events
