# Supplementary material for: The efficacy of small molecule anti-angiogenic drugs in previously treated Thymic carcinoma
Source: BMC Cancer. 2023 Jan 5;23:16. doi: 10.1186/s12885-022-10448-z (PMC9817249; doi:10.1186/s12885-022-10448-z)
Supplement: Supplementary file 1 — Additional file 1: Supplementary Table 1. Univariate COX regression analysis of PFS and OS. [file 12885_2022_10448_MOESM1_ESM.docx]

|  | **Univariate of PFS** | | **Univariate of OS** | |
| --- | --- | --- | --- | --- |
|  | P value | HR 95% CI | P value | HR 95% CI |
| Gender (Male vs Female) | 0.615 | 1.313(0.453-3.805) | 0.784 | 0.827(0.212-3.221) |
| Age (<60 vs ≥60) | 0.657 | 0.777(0.255-2.368) | 0.193 | 2.791(0.595-13.084) |
| Smoking (Never vs. Current/Former） | 0.834 | 1.256(0.423-3.730) | 0.832 | 0.839(0.166-4.240) |
| PS score (0 vs 1) | 0.026 | 4.531(1.199-7.161) | 0.359 | 2.102(0.430-10.277) |
| Lung metastasis (No vs Yes) | 0.725 | 1.473(0.171-12.690) | 0.120 | 2.878(0.758-10.926) |
| Liver metastasis (No vs Yes) | 0.311 | 0.559(0.181-1.724) | 0.343 | 0.351(0.040-3.060) |
| Bone metastasis (No vs Yes) | 0.960 | 0.972(0.321-2.942) | 0.531 | 0.640(0.158-2.585) |
| HFS (No vs Yes) | 0.327 | 0.577(0.192-1.731) | 0.684 | 0.753(0.192-2.948) |
| hypertension (No vs Yes) | 0.384 | 0.592(0.182-1.928) | 0.547 | 0.647(0.157-2.665) |
| *HFS,* hand-foot syndrome | | | | |

**Supplementary table 1.** Univariate COX regression analysis of PFS and OS
